# Author Index for Volume105

**DOI:** 10.1038/bjc.2011.539

**Published:** 2011-12-06

Aas, T 9

Abbod, MF 931

Abdirad, A 38

Abe, H 1615

Abenhardt, W 206

Abia, D 1600

Abramovitz, M 1574

Abulí, A 870

Abulrob, A 1697

Adelaide, J 304

Adhim, Z 393

Adwan, H 288

Adzovic, S 1741

Afanasyeva, Y 1458

Affolter, A 353

Agerbæk, M 1379

Agnese, DM 1023

Ahmad, AS 1795

Ahmad, I 1362

Ahmad, T 353

Ahnen, DJ 162

Aitken, J 1039

Akashi-Tanaka, S 698

Åkesson, A 441

Akiba, S 38

Akiyama, F 1197

Akslen, LA 9

al-Abany, M 737

Albain, KS 796

Albanell, J 814

Albertsen, PC 931

Albregtsen, F 1218

Alcaraz, A 1600

Alecci, C 1542

Alevronta, E 737

Alexandre, J 1640

Ålgars, A 255

Alibhai, SMH 606

Ali-Fehmi, R 493

Alitalo, K 1346

Allan, H 903

Allen, NE 1436

Allen, V 340

Al-Mahmood, S 1002

Aloyz, R 1342

Alsaker, MDK 731

Alston, R 177

Alyasiri, A 1654

Ambros, I 1940

Ambros, P 1940

Anai, S 1191

Andersen, CL 552

Anderson, J 467

Andersson, LC 1346

Andersson, S-O 1061

André, F 847

Andrén, O 1061

Andreola, F 1370

Andreotti, G 1424

Andreu, M 870

Andrulis, I 1934

Ang, YS 124

Angleitner-Boubenizek, L 1144

Anton-Culver, H 1934

Antonenkova, N 1934

Antonetti, R 1183

Anu, R 513

Anwar, M 38

Aoki, M 824

Aomatsu, K 1210

Apetoh, L 366

Apicella, C 1934

Appleby, VA 575

Arai, E 1839

Araki, K 1885

Arao, T 407, 1210

Arbyn, M 877

Ardanaz, E 1436

Arenas, RB 1203

Arendzen, AJ 312

Argimon, JM 753

Argüello, L 870

Armstrong, G 766

Armstrong, K 1362

Arndt, V 1158

Arnoletti, JP 523

Arnould, L 366

Arpí, O 814

Arriola, E 814

Arslan, AA 1458

Arumí-Uría, M 814

Ashcroft, L 22

Ashworth, A 911

Assaraf, YG 1542

Asselain, B 1480

Atkins, L 18, 1474

Atkins, MB 112

Auger, N 1940

Ault, K 28

Auvinen, A 1069

Åvall-Lundqvist, E 737

Awada, A 1726

Ayala de la Peña, F 612

Ayhan, A 890

Azodi, M 1176

Baade, PD 1039, 1076

Baccarelli, A 1772

Bacon, M 360

Baffy, G 469

Baglietto, L 1934

Bai, L-Y 1419

Baiget, M 53

Bailey, HD 1409

Bakken, K 1436

Balakrishnan, A 1825

Baldwin, DR 746

Ballinger, R 1260

Balteskard, L 1719

Bamia, C 1436

Bando, H 403

Bane, F 118

Baracos, VE 1244

Barbault, A 640

Barber, B 1495

Barlesi, F 1123

Baron, JA 162

Barone, G 586

Baron-Lühr, B 673

Barrett, HL 1487

Bartrar, CR 1934

Barwick, BG 1574

Basta, NO 1402

Basu, S 1920

Batus, M 1920

Bauer, S 263

Bautista, OM 28

Beale, G 372

Bearss, D 1563

Beavan, J 918

Becker, N 1768

Beckmann, MW 1934

Beiske, K 1940

Béliveau, R 1697

Bellone, S 1176

Bellosillo, B 814

Belting, M 666

Bénard, J 1352, 1940

Benavente, Y 1768

Benítez, J 1934

Bennett, L 1495

Bennouna, J 1646

Bentley, B 1203

Bentley, J 1144

Berek, JS 890

Bergen, AW 320

Berger, A 1593

Berger, MR 288

Bergmann, F 288

Bermudo, R 1600

Bernard, L 1934

Bernhard, H 596

Bernhardt, P 682, 1468

Berrino, F 1458

Berrino, L 382

Bertazzi, PA 320

Bertrand, Y 1697

Bertucci, F 304

Bessa, X 870

Bhandari, P 239

Bhatt, RS 112

Bichev, D 505

Bieblová, J 1533

Bieniasz, M 1512

Bigelow, C 1203

Billingham, LJ 628

Billiot, F 847

Bingham, V 1313

Birch, J 177

Birkenkamp-Demtröder, K 552, 1379

Birnbaum, D 304

Bissinger, O 1864

Biswas-Baldwin, N 618

Bito, T 393

Bjarnason, GA 1741

Blanco, D 1608

Blann, AD 1252

Blau, I 505

Bliss, JM 1260

Böckelman, C 989

Bofetta, P 1768

Bogdanova, N 1934

Bonanni, B 1934

Bonensea, J 304

Bongaerts, M 1002

Bongers, EMHF 1912

Bonnetain, F 1480

Bono, P 1346

Bonomi, PD 1920

Börger, V 961

Borgulya, G 185

Borràs, JM 753

Borre, M 1379

Børresen-Dale, A-L 9

Bose, D 1759

Bosserhoff, A-K 231

Bota, S 1123

Botti, G 1030

Bouganim, N 1342

Boulghourjian, A 272

Bourgeois, H 1144

Bourhis, J 618

Bousquet, G 1640

Bouzyk, M 1574

Bown, N 1940

Brabin, L 177

Braem, MGM 1436

Braga, M 428

Braicu, EI 890, 1593, 1818

Braig, S 231

Brain, E 1480

Branstetter, D 1830

Bratton, DJ 1451

Bray, F 723

Breithaupt, K 505

Bremer, M 1934

Brems Eskildsen, A-S 1379

Brennan, P 1768, 1934

Brenner, H 1158

Brezovich, I 640

Brichard, B 1940

Bridgewater, J 1646

Briest, S 445

Brisson, M-L 1342

Broadhead, ML 1503

Brock, IW 1934

Broeders, MJM 594

Broeks, A 1934

Brown, A 445

Brown, C 360, 1144

Brown, JM 194

Brown, SR 194

Browne, L 272

Brozyna, AA 1874

Bruin, SC 281

Bruland, O 1719

Brüning, T 1934

Brunner, TB 628

Bryan, J 28

Bubeník, J 1533

Buchanan, DD 162

Buck, K 1151

Buckingham, L 1920

Buczacki, S 1253

Buechner, J 296

Bueno-de-Mesquita, HB 1436

Bujanda, L 870

Bujold, R 534

Bulten, J 1279

Bünemann, E 961

Buonaccorsi, GA 139

Burczynski, ME 246

Burdach, S 596

Burden, RE 1487

Burdett, S 1107

Burges, A 1144

Burgess, C 18

Burnet, NG 628

Burwinkel, B 1934

Busch, DH 596

Busch, J 1635

Busson, P 1352

Buter, J 44

Bützow, R 989

Buyse, M 194

Buza, N 1176

Byrd, PJ 586

Byrne, C 118

Cadron, I 1593

Cafferty, F 1107

Calvo, A 1608

Câmara, JS 1894

Camargo, MC 38

Cameron, D 1260

Campbell, FC 1313

Campbell, PT 162

Campone, M 1646

Cañadas, I 814

Cañete, A 89, 1940

Canteras, M 612

Cantor, A 640

Cantwell, MM 13

Cao, QJ 1203

Caporaso, NE 320

Capp, A 272

Carew, JS 1563

Carey, FA 246

Carmona-Bayonas, A 612

Carozza, SE 1396

Carpagnano, F 1183

Carpagnano, GE 1183

Carpén, O 255

Carpenter, LM 1783

Carracedo, A 870

Carracedo, C 618

Carrara, L 1176

Carrascal, E 38

Carreño, R 870

Carson III, WE 1023

Carvajal-Carmona, L 870

Casabonne, D 1768

Casado, E 1646

Cascone, T 382

Casey, G 162

Cassidy, J 58

Castaigne, J-P 1697

Castel, V 89, 1940

Castells, A 870

Castellsagué, X 28

Castellví-Bel, S 870

Castro, E 1230

Catena, R 1608

Catto, JWF 931

Catzavelos, C 1574

Cauda, R 513

Cerella, C 221

Cerhan, J 1934

Cerman Jr, J 1646

Cersovsky, SB 602

Chaiwun, B 1224

Challberg, J 22

Chammas, MC 640

Chan, CS 628

Chan, D 842

Chan, JK 1137

Chan, K-K 542

Chan, SL 658

Chang, ET 1776

Chang-Claude, J 1151, 1158, 1934

Chanock, S 1934

Chao, D 346, 353

Chapman, MH 1370

Chateauvieux, S 221

Chauchereau, A 847

Chaudhuri, G 428

Cheema, M 466

Chemidlin, J 44

Chen, CC 1235

Chen, C-Y 975, 1927

Chen, S-W 1419

Chen, T 372

Chen, X 1934

Chen, Y 945, 1512

Chen, Y-C 975

Chenevix-Trench, G 1934

Cheng, C-L 658, 975

Cheng, S 606

Chester, J 766

Cheung, S 139

Chevreau, C 353

Chi, DS 890

Chi, J-T 9

Chiba, T 1693

Chinegwundoh, F 481

Chiu, S 1388

Chiyomaru, T 833

Choong, PFM 1503

Chou, W-C 975, 1927

Chouaid, C 1123

Chow, EJ 1396

Chow, PKH 945

Chow, W-H 1096, 1443, 1772

Choy, G 1563

Christiansen, CF 881

Chu, C-N 1419

Chu, QS-C 938

Ciafrè, SA 1352

Ciardiello, F 382

Ciesielski, MJ 1512

Cipok, M 1708

Clamp, AR 139

Clark, J 1023

Clarke, AR 649

Clarke, S 58

Clarkson, A 1370

Clavel-Chapelon, F 1436

Clayton, R 22

Clementz, AG 796

Cliby, WA 372

Clifford, SC 575

Cocco, E 1176

Cocco, PL 1768

Coggon, D 1054

Cohen, V 1342

Cole, SR 602

Coleman, N 575

Coleman, R 189

Colin, S 1002

Colley, HE 1582

Collinson, F 194

Colt, JS 1096, 1772

Combaret, V 1352, 1940

Compton, N 618

Concin, N 1593

Conrad, H 596

Cook, N 340

Coon, J 1920

Coral, S 327

Cormio, G 890

Corre, R 1123

Corrie, P 353

Corvalan, AH 38

Costa, FP 640

Couch, FJ 1934

Coudert, B 366

Coupland, C 452

Couturier, J 1940

Cox, A 1934

Craft, AW 1402

Cree, IA 239

Creoff, E 1002

Croce, CM 1023

Cross, AJ 1096

Cross, SS 1934

Croucher, R 925

Crowther, D 246

Cummins, R 124

Cupissol, D 618

Currie, J-C 1697

Curtin, NJ 372, 1114

Dadaev, T 1230

Daemen, T 93

Dahl, O 1719

D’Aiuto, E 382

Daladimos, T 897

Dalgleish, AG 687, 1554

Dallas, N 1759

Dalton, SO 1042

Damms, P 340

Daniel, CR 1096

Danielsen, HE 1218

D’Antonio, A 1030

Dao, KM 760

D’Armiento, J 1615

Darvasi, A 864

Dasgupta, P 1039

Dasgupta, S 1295

Dass, CR 1503

Davies, EA 1049

Davies, EG 586

Davies, RJ 1253

Davis, FG 1096, 1772

Davis, M 931

Day, FL 498

de Bernardi, B 1940

de Bock, GH 93

de Boer, CJ 346

de Bont, ESJM 1856

De Donatis, GM 513

de Groot, KA 281

de Groote, ML 312

de Hullu, JA 1279

de Jong, MM 1912

de Jonge, HJM 1856

de Jonge, MJA 938

de Klerk, NH 1409

de Koning, HJ 1082

de Lacerda, A 1940

De Luca, A 1030

De Marzo, AM 602

de Mont-Serrat, H 1640

de Oliveira, AC 640

De Palma, R 382

De Raucourt, D 618

de Sanjosé, S 1768

de Souza Rocha, M 640

De Velasco, MA 1210

de Winton, E 498

Dearnaley, D 467

deBlacam, C 118

Decker, T 206

Dees, J 200

Deevi, R 1313

Defferrari, R 1940

del Campo, JM 618

del Rio, E 53

Delaney, G 272

Delattre, O 1940

Delhoume, JY 1123

Delord, J-P 1646

Delva, R 1480

Demeule, M 1697

den Dunnen, WFA 1856

den Heeten, GJ 594

den Hoed, CM 200

Denkert, C 1818

Derdak, Z 469

Deshmukh, NS 931

Desmarais, G 534

Dessen, P 1352

Destro, A 1542

Deutsch, VR 1708

Dey, P 340

Dhillon, PK 723

di Cataldo, A 1940

Di Fiore, F 1811

Di Mauro, M 1663

Di Taranto, A 1183

Díaz-Rubio, E 58

DiCarlo, B 760

Diederich, M 221

Dietz, A 445

Dietzfelbinger, H 206

Dikshit, R 723

Dinjens, W 854

Diorio, C 606

Discacciati, A 1061

Dite, GS 1934

Dittmer, D 320

Dogan, Y 505

Doi, T 403

Doi, Y 996

Dolecek, TA 1414

Dolznig, H 263

Domanitskaya, NV 74

Dómine, M 814

Donadello, N 360

Donders, R 594

Dong, LM 1096

Donovan, JL 931

Dörk, T 1934

dos Santos Silva, I 1934

Dossus, L 1436

Douc-Rasy, S 1352

Douillard, J-Y 1495

Downey, P 124, 1669

Downie, L 618

Downie, P 1409

Dowty, JG 162

Draisma, G 1082

Drew, AK 1166

Drusch, F 847

du Bois, A 890

Dujon, C 1123

Duku, M 239

Dunberger, G 737

Dunn, JM 1218

Dunning, AM 1934

Dvorak, A 1654

Dvorak, J 1646

Dyrskjøt, L 1379

Dziadziuszko, R 1

Easton, DF 467, 586, 1230, 1934

Eberhard, J 666

Eckersley, B 22

Eckert, K 445

Eckhardt, SG 1830

Edmondson, RJ 372

Edwards, J 1362

Edwards, S 1230

Eeles, RA 467, 1230

Eger, A 263

Einvik, C 296

Eisen, T 353

Eizuru, Y 38

Ekici, AB 1934

Elashoff, R 760

El-Hariry, I 618

El-Khoueiry, A 44

Elliott, DJ 602

Elliott, G 1934

Ellis, LM 1759

Ellis, P 1260

El-Serag, H 154

El-Tanani, M 542

Endo, M 1267

Engel, P 1436

English, DR 162, 438

Enokida, H 833

Erichsen, R 881

Espinàs, JA 753

Espitia, CM 1563

Esquivel, JA 1563

Esumi, H 403

Ethier, MC 606

Etminan, N 961

Evans, AJ 1741

Evans, DG 22

Eyol, E 288

Fairchild, ET 1023

Faithfull, S 903

Faivre, S 1640

Falchero, L 1123

Faller, W 565

Fallowfield, L 189, 1260

Fan, F 1759

Farace, F 847, 1338

Farnoushi, Y 1708

Farshid, G 1669

Fasching, PA 1934

Fatehi, D 1697

Fatehullah, A 1313

Faull, C 918

Fayers, PM 1244

Fearon, K 1244

Fedirko, V 1436

Feltquate, D 44

Fennell, DA 1, 542

Fenner, M 1635

Fernández, PL 1600

Fernández-Rozadilla, C 870

Ferrario, C 1342

Ferrero, A 1144

Ferris, D 28

Feuerstein, G 246

Feychting, M 1069

Ficorella, C 1663

Fidler, MJ 1920

Figer, A 58

Findlay, M 945

Finetti, P 304

Fischer von Weikersthal, L 206

Fisher, D 1512

Fizazi, K 847

Fladvad, T 1244

Flægstad, T 296

Flesch-Janys, D 1151

Fleshman, JW 1654

Fluge, Ø 1719

Foley, G 177

Font, R 753

Forbes, LJL 18, 1474

Ford, S 44

Foretova, L 1768

Forssell-Aronsson, E 682, 1468

Foschino-Barbaro, MP 1183

Fotopoulou, C 890, 1818

Foulkes, WD 864

Fournier, A 1436

Fowler, DW 687

Fox, C 272

Fox, EE 1396

Fratini, S 1663

Fratta, E 327

Frau, A 1726

Fredericksen, Z 1934

Frederiksen, BL 1042

Free, CM 746

Friedlander, M 360, 1144

Friess, H 288

Fristrup, N 1379

Fritschi, L 1076, 1409

Frolov, A 523

Frøslev, T 881, 1776

Frost, AR 523

Frost, C 1451

Fuchs, J 212

Fuchshofer, R 231

Fujie, H 1302

Fujimoto, K 1191

Fujimura, L 833

Fujisaka, Y 1267

Fujisawa, M 393

Fujita, Y 1210

Fujiwara, H 104

Fukuda, H 1615

Fukuda, T 1273

Fukuma, D 1322

Fumoleau, P 366, 1480

Furukawa, H 131

Fuse, N 403

Futamura, N 1839

Fuyuhiro, Y 996

Gaber, A 666

Gabrieau, V 1934

Gabril, M 1741

Galasso, R 1436

Galbraith, S 44

Galibert, M-D 1726

Gallagher, D 864

Gallagher, WM 565

Gallinger, S 162

Gandhi, M 945

Ganser, A 1635

Gao, Y-T 1424, 1443

García, AM 870

Garcia-Closas, M 1934

García-Cruz, E 1600

Garrett, CR 44

Gattolliat, C-H 1352

Gaur, P 1759

Gauthier, M 1480

Gaydos, CA 602

Gebski, V 360, 1144

Geisler, S 9

George, D 112

Georges, R 288

Geraci, M 177

Gerdes, J 673

Gerrard, M 1940

Ghanem, G 1726

Ghiringhelli, F 366

Giancola, F 1542

Gianoncelli, L 1542

Gibbens, I 353

Gibson, L 1934

Giese, NA 288

Giesinger, J 445

Giessen, C 206

Giessrigl, B 263

Gijezen, LM 1912

Gil, M 1512

Gilberg, F 58

Giles, FJ 1563

Giles, GG 438, 1934

Gill, A 1370

Gill, PG 1669

Gille, JJP 1912

Gillet, J-P 513

Gillis, AJM 854

Giovannetti, E 1542

Giovannucci, E 65

Giráldez, MD 870

Girschik, J 1076

Glass, DC 1409

Glendon, G 1934

Glover, JA 13

Glynn, RW 1625

Goepel, JR 931

Goh, C 1230

Goldgar, D 1230

Goldmann, T 673

Goldstein, AM 320

Goldstein, RS 1708

Goldwasser, F 1640

Gómez, J 612

Gomez-Pozo, B 1402

Gonzalez, R 346

González-Billalabeitia, E 612

González-Neira, A 1934

Gonzalo, V 870

Gonzálvez, ML 612

Gooden, MJM 93

Gore, ME 346, 353

Gormley, JA 1487

Gornick, MC 562

Gosney, M 1260

Gottesman, MM 513

Govindarajan, S 1430

Graham, PH 272

Gram, IT 1436

Grande, E 814

Grant-Pearce, C 340

Graubard, BI 1096, 1772

Gravett, AM 687

Gregory, WM 194

Greig, D 1814

Greillier, L 1

Gremel, G 565

Grieser, C 505

Griffin, CL 884

Griffiths, DF 931

Grignol, V 1023

Grigor, KM 931

Grigorieva, EV 74

Grigull, J 1741

Grills, C 542

Grip, M 1934

Groshen, S 1224

Grothoff, M 505

Gruber, SB 562

Grünwald, V 1635

Grusch, M 263

Gu, G 1905

Gualberto, A 1467

Guerrero-Preston, R 1295

Guertler, U 1554

Guha, S 864

Guilmain, W 1002

Gujral, DM 1804

Gulley, ML 320

Gulmann, C 124

Guo, B 124

Gurney, H 360

Gustavsson, I 694

Gutzmer, R 346

Guy, M 467, 1230

Guzzo, F 1176

Gyllensten, U 694

Haberl, C 206

Hager, G 1593

Hagi, T 778

Haglund, CH 989, 1346

Hahn, S 552

Haile, RW 162

Hait, W 346

Hakama, M 1388

Hall, A 1230

Hall, AL 467

Hallmans, G 1458

Halloran, SP 475

Hamann, U 1934

Hamasaki, H 824

Hamasaki, M 824

Hamdy, FC 931

Hamel, N 864

Hamilton, M 938

Hämmerle, M 263

Han, C 1252

Han, T-Q 1424

Hanazawa, T 833

Hanchett, N 170

Handley, M 513

Hänggi, D 961

Hanna, R 493

Hansen, L 1436

Hantusch, B 263

Hao, Z 38

Harabuchi, Y 833

Hara-Miyauchi, C 1615

Harries, M 353

Harrington, KJ 618, 628

Harris, AL 1252

Harriss, D 467

Hart, A 340

Harter, P 890

Hartikainen, J 1934

Hasebe, T 698

Hasegawa, T 996, 1522

Hass, HG 206

Haste, F 1474

Haug, BH 296

Haupt, RM 28

Hauss, J 445

Hayes, W 44

He, Y 281

Hearnden, V 1582

Hecht, JR 760

Heederik, D 1054

Hegarty, SM 565, 1487

Heijnsdijk, EAM 1082

Hein, A 1934

Hein, R 1934

Heinemann, V 206

Heinz, J 1151

Hemmes, A 989

Hemming, J 911

Hendriks, JCM 1279

Hendrix, MJC 1030

Heneghan, C 475

Henningson, M 1676

Henriksen, JR 296

Herbst, RS 1830

Heriot, A 498

Hermann, GG 1379

Herrera-Goepfert, R 38

Heslin, MJ 523

Hewitson, P 475

Heymach, JV 112, 382

Hickish, T 1788

Hicks, RJ 498

Hietala, M 1676

Hill, ADK 118

Hill, R 1012

Hillemanns, P 1934

Hinten, F 1279

Hippisley-Cox, J 452

Hirakawa, K 996, 1522, 1750

Hiraki, A 1322

Hirao, Y 1191

Hiraoka, N 131

Hirsch, FR 814

Hirte, H 884, 1144

Hitt, R 618

Hixon, ML 1467

Hjellvik, V 157

Ho, P 346

Ho, S-M 65

Hoang, H-H 945

Hoffmeister, M 1158

Hofmann, JN 1772

Hofmeister, D 445

Hofstetter, G 1593

Högberg, T 1144

Hogg, A 498

Hoggard, E 1392

Hojo, T 698

Holland, PM 1830

Holland, R 594

Holm, PS 1864

Holm, T 1392

Holman, CDJ 1089

Holmberg, L 170

Honey, RJ 1741

Hoogland, AM 854

Hoover, RN 1934

Hopkins, A 372

Hopper, JL 162, 438, 1934

Hopwood, P 22

Horai, T 1131

Horel, S 1396

Horii, R 1197

Horiike, A 1131

Hoskin, P 628

Hoskins, JM 1654

Hotta, K 1191

Hou, H-A 975, 1927

Houlston, RS 870

Houweling, AC 1912

Howell, A 22

Howell, D 1684

Hoxha, M 1772

Hsieh, AC 329

Hsing, AW 1424

Hsu, CY 1419

Hsu, M-Y 1030

Hsu, S-C 975

Huang, C-F 1927

Huang, C-P 1830

Huang, S-Y 975

Hubbard, RB 746

Huerta, JM 1436

Hughes, CM 13

Hughes, E 118

Hui, P 1176

Humphreys, MK 1934

Hunter, DJ 1934

Hurley, MA 1054

Huttary, N 263

Hveem, T 1218

Iacopetta, B 658

Ichikawa, D 104, 1733

Id Boufker, H 1726

Idahl, A 1436, 1458

Iida, K 420

Iitaka, D 104

Ikoma, H 1733

Illidge, T 628

Imam, SA 1224

Immonen-Räihä, P 1388

Indrová, M 1533

Ingvar, C 1676

Ioannou, N 1554

Isaacs, WB 602

Ishiguro, H 1693

Ishiguro, N 1839

Ishikawa, M 420

Islam, SS 925

Iwabuchi, Y 212

Iwasaki, H 824

Iwasaki, M 131, 698

Iwase, T 1197

Iwata, K 938

Izatt, L 586

Jack, A 1684

Jack, RH 1049

Jackson, A 139

Jacobs, ET 162

Jacobsen, E 1042

Jacobsen, J 881

Jacobson, KK 1920

Jacquemier, J 304

Jacques, N 847

Jagan, I 1313

Jäger, W 263

Jakobsen, S 1850

James, MG 353

James, PW 1402

Jan, H 1708

Janjetovic, Z 1874

Jankowski, JAZ 1105

Janoueix-Lerosey, I 1940

Jansen, L 1158

Janszky, I 731

Jaspars, EH 1912

Javle, M 1114

Jayson, GC 139

Jenkins, MA 162

Jennings, VA 787

Jenster, G 854

Jernström, H 1676

Jewell, SS 1920

Jewett, MAS 1741

Jeyapalan, JN 575

Jhuang, J-Y 975

Ji, B-T 1443

Jiang, R 890

Jiao, W 1905

Jimenez, M 1480

Jin, M-Y 945

Jirström, K 666, 1436

Job, B 1352

Joensuu, H 1346

Joffe, J 766

Johannesma, PC 1912

Johansen, C 1042, 1069

Johansson, J-E 1061

Johansson, K-A 737

Johansson, ME 666

John, EM 1934

Johnson, KJ 1396

Johnson, L 1260

Johnson, N 1934

Johnsson, A 666

Johnston, JA 1487

Johnston, PG 1487

Jones, A 189

Jones, AV 1582

Jones, ME 911

Jonker, MA 1912

Jørgensen, KJ 592

Joske, D 1076

Jouary, T 353

Journe, F 1726

Jover, R 870

Joyce, C 565

Jozwicki, W 1874

Jubitz, N 1864

Jukkola-Vuorinen, A 1934

Julin, B 441

Jullian, H 1123

Junglas, B 231

Kaaks, R 1436

Kaasa, S 1244

Kacher, JE 1023

Kai, J 918

Kaiser, R 1640

Kajiyama, H 1288

Kalinoglou, N 897

Kalna, G 1362

Kalt, R 263

Kampen, KR 1856

Kamps, WA 1856

Kanai, M 1693

Kaneda, H 1210

Kaneko, K 403

Kang, D 1934

Kang, Y 1805

Kapp, DS 1137

Kappauf, H 206

Karabelis, A 897

Karpa, VI 1023

Kasahara, K 1131

Kashiwagi, S 996

Kashuba, VI 74

Kasper, EM 1235

Katagiri, A 420

Kataja, V 1934

Katayama, A 833

Kato, Y 996

Kattoor, J 38

Kauhava, L 1388

Kawabata, M 393

Kawahara, K 1322

Kawai, M 1288

Kawakami, K 833

Kawamura, K 1302

Kay, EW 1487

Kay, S 1708

Kazemier, HG 312

Kearsley, JH 272

Keidar, M 1295

Keiding, S 1850

Keld, R 124

Kelly, KR 1563

Kelly, RS 983

Kerin, MJ 1934

Kerr, DJ 870

Key, TJ 1436

Khan, OA 1252

Khaw, K-T 1436

Kheirandish, P 481

Khella, HWZ 1741

Kiet, TK 1137

Kikkawa, F 1288

Kikkawa, N 833

Kikuchi, E 1331

Kilburn, LS 1260

Kilday, JP 575

Kim, HH 413

Kim, T-K 1874

Kim, WH 38, 413

Kimata, K 1839

Kimura, K 1197

Kinoshita, T 698

Kirchhoff, T 864

Kirichek, O 1252

Kirkwood, JM 773

Kitano, T 1693

Kitchener, HC 177, 983

Klaeboe, L 1069

Kleeff, J 288

Klein, RJ 864

Klein, S 206

Klemi, P 1388

Klepstad, P 1244

Klijn, C 281

Knight, JA 1934

Ko, B-S 975

Ko, YD 1934

Kobayahi, T 1885

Kobayashi, H 420, 1302

Kodani, M 1574

Koenig, KL 1458

Koga, K 824

Koh, W-P 1430

Kojima, M 403

Kojima, T 403

Kokubu, A 131

Kolk, A 1864

Kollia, G 44

Komatsu, S 104, 1733

Konishi, H 104, 1733

Konishi, N 1191

Kono, T 131

Koriyama, C 38

Korse, CM 1173

Kosaka, T 1331

Kosel, M 1934

Koshikawa, N 824

Koshiol, J 320

Koshy, M 1414

Koski, S 58

Kosma, V-M 1934

Kosmas, C 897

Kossenkov, AV 523

Kosuga, T 104

Kosuge, T 131

Kote-Jarai, Z 467, 1230

Koutelou, A 1183

Kozawa, E 1839

Kozbor, D 1512

Koziol, JA 599

Kozono, D 1235

Kremer, M 1864

Kretschy, N 263

Kretzschmar, A 505

Krieger, S 263

Krogh V 1458

Kroll, ME 1783

Krupitza, G 263

Kubo, N 1885

Kucharzewska, P 666

Kudo, K 1131, 1210

Kudoh, S 1267

Kugler, C 673

Kuhnt, S 445

Kuipers, EJ 200

Kulesza, P 523

Kumar, S 493

Kunkler, I 189

Kuo, Y-Y 1927

Kurkure, AP 723

Kuster, N 640

Kuwano, H 1885

Kyle, S 372

Labo, N 1768

Lacroix, L 847

Ladenstein, R 1940

Ladoire, S 366

Lagergren, J 154

Lakbir, D 961

Lakshmi, BS 953

Lalloo, F 22

Lambaudie, E 304

Lamura, L 1030

Lanas, A 870

Landi, MT 320

Lane, JA 931

Lang, Z 346

Langley, RE 1107

Langmár, Z 185

Langton, JM 1166

Lanigan, F 565

Laplanche, A 847

Lappin, TR 542

Larjavaara, S 1069

Larrañaga, N 1436

Larsson, A 666

Larzabal, L 1608

Lassus, H 989

Last, JI 586

Laurent, G 1726

Lavelle, M 565

Lawson, D 346

Layburn, J 1474

Le Marchand, L 162

Le Moulec, S 847

Le Teuff, G 1352

Le Tourneau, C 1640

LeCaer, H 1123

Lecharpentier, A 1338

Ledermann, JA 884

Lee, CK 360, 1144

Lee, F-Y 1927

Lee, HE 413

Lee, HS 413

Lee, K-M 393

Lee, MC 1927

Lee, PWK 1012

Lee, S-H 575

Leeson, S 877

Leffers, N 93

Legrand, E 1002

Lehman, A 1023

Leighl, NB 1

Leminen, A 989

Lencz, T 864

Lenner, P 1458

Lenters, V 1054

Leon, LG 1542

Leonard, R 189, 1260

Leong, T 498

Leongamornlert, D 1230

Lesinski, GB 1023

Leter, EM 1912

Leung, HY 1362

Levy, C 1480

Lewis, WG 842

Leyland-Jones, B 1574

Li, D 1905

Li, H 212

Li, H-L 1443

Li, P -K 212

Li, Q 1302

Li, W 1905

Li, Z 1574

Liebler, DC 1759

Liefers, G -J 281

Liles, JS 523

Lim, C-H 945

Limburg, PJ 1105

Lin, C-W 975

Lin, J 212

Lin, K 1176

Lin, L 212

Lin, L-S 760

Lind, H 737

Lindblom, A 1934

Lindell, M 337, 694

Linden, W 1814

Linder, N 1346

Lindor, NM 162

Link, E 498

Linkens, DA 931

Linnoila, I 320

Linseisen, J 1151

Linsell, L 18

Lintunen, M 255

Lipton, L 162

Lissowska, J 1934

Liu, C-Y 1927

Liu, H 1905

Liu, J-F 467

Liu, M 1458

Liu, M-C 1927

Liu, WM 687

Liu, Y 212

Llor, X 870

Lobo, R 945

Lokanatha, D 618

Løkke, C 296

Lønning, PE 9

Looijenga, LHJ 854

Lopez-Vilariño, JA 814

Lophatananon, A 467

Loquai, C 346

Lord, S 360, 1144

Lorenzi, E 1542

Lorigan, P 353

LoRusso, P 44

Louhimo, J 1346

Lovat, LB 1218

Lüchtenborg, M 170

Lucia, MB 513

Lukanova, A 1436, 1458

Luna, J 28

Lund, E 1436

Lundin, E 1458

Lundin, J 1346

Lundin, KB 1676

Lundin, M 1346

Lynch, C 498

Macdougall, J 628

Machado, MCC 640

Machin, D 945

Mackay, RI 628

MacKenzie, R 1814

Mackintosh, D 1554

MacNeil, S 1582

Macrae, FA 162

Mader, R 263

Maders, F 139

Madlener, S 263

Madsen, HHT 1850

Maenpaa, J 360

Maghnouj, A 552

Magro, CM 1023

Mahalingam, D 1563

Mahdi, H 493

Mahmud, N 1230

Mahner, S 1593

Maio, M 327

Major, P 44

Makris, A 1260

Malila, N 1388

Mallary, S 28

Mallick, P 65

Mancini, M 1663

Manjer, J 1436

Mannermaa, A 1934

Manning, JT 438

Manoukian, S 1934

Mans, S 970

Mansi, J 1260

Mansilla, F 552, 1379

Mant, D 475

Mantwill, K 1864

Marais, R 353

Marcato, P 1012

Marcuello, E 53

Margaryan, N 1030

Margolin, S 1934

Mari, E 1030

Marian, B 263

Marincola, FM 320

Marlow, LAV 486

Maroudias, N 618

Marques, B 1940

Marshall, C 1814

Marshall, D 346

Marshall, J 44

Martens, J 854

Marth, C 1593

Martin, AL 1480

Martin, F 366

Martinelli, D 1183

Martinelli, E 382

Martínez, ME 162

Martin-Hirsch, P 877

Martino, J 606

Marwitz, S 673

Masala, G 1436

Mason, M 1628

Massard, C 847

Massuger, LFAG 1279

Matsumoto, K 1210

Matsumoto, S 1693

Matsuoka, J 996

Matsuoka, T 393, 1750

Matsuzaki, T 1522

Mattarollo, SR 778

Matthews, CE 1443

Mattsson, F 154

Maughan, TS 628, 649

Mauriac, L 1480

May, E 1352

Mayer, NJ 931

Maynadie, M 1768

Mayo, C 606

Mazhar, D 766

Mazurak, VC 1469

Mazzocco, K 1940

Mbisa, G 1768

McCarthy, BJ 1414, 1772

McCarthy, S 938

McDermott, EW 565

McElvenny, DM 1054

McIlroy, M 118

McKay, J 1934

McKenna, WG 628

McLaughlin, CC 1396

McLaughlin, PMJ 312

McLean, CA 1934

McLeod, HL 1654

McNally, RJQ 1402

McNeil, CM 272

McShane, LM 320

Mead, R 239

Medina, EC 1563

Meeke, K 796

Meeuwsen-de Boer, TGJ 1856

Mehrara, E 682, 1468

Meier, W 360

Meindl, A 1934

Meirelles, R 640

Melcher, AA 787

Meldgaard, P 1850

Melichar, B 1646

Mellish, L 1166

Memon, AA 1850

Ménager, C 1811

Menéndez, S 814

Mengele, K 1864

Menko, FH 1912

Metcalfe, C 931

Meurice, G 1352

Meyer, TE 1424

Michael, M 498

Michel, P 1811

Michon, J 1940

Middleton, MR 1252

Midwinter, D 618

Miele, G 246

Miele, L 796

Mignot, G 366

Mikhaylina, A 523

Mikolajewska-Hanclich, I 281

Mikulits, W 263

Mikyšková, R 1533

Millar, EKA 272

Miller Jr, WH 1342

Milne, E 1409

Milne, RL 1934

Miron, A 1934

Mirza, KA 83

Misset, JL 1640

Mistry, M 1795

Mita, AC 938

Mita, MM 938

Mittermüller, J 206

Miyajima, A 1331

Miyamoto, M 131

Miyauchi, E 1131

Miyazaki, K 420

Miyazaki, Y 1331

Mizuno, M 1288

Mochizuki, S 1615

Modjtahedi, H 1554

Mohamed Noor, DA 575

Mohr, P 346

Mokliatchouk, O 44

Møller, H 170, 1049

Monsonego, J 28

Montegriffo, E 353

Montuenga, LM 1608

Moodie, K 498

Moosmann, N 206

Moreno, CS 1574

Moreno, V 562

Morgan, D 640

Morgillo, F 382

Mori, Y 1693

Morimura, R 104, 1733

Morreau, H 281

Morris, RT 493

Morrison, LE 1920

Moschos, S 773

Moschovis, D 897

Moss, SM 594, 983

Mosseri, V 1940

Mostovich, LA 74

Motsinger-Reif, AA 1654

Mou, C-H 1419

Mourtzakis, M 1469

Mouw, T 1436

Mozos, A 1600

Mueller, BA 1396

Mueller, BU 970

Muir, K-R 467

Muller, DC 438

Muller, M 1173

Müller-Spahn, C 596

Mulligan, AM 1934

Munkarah, AR 493

Munksgaard, PP 1379

Muñoz, J 870

Muñoz, N 28

Munzer, C 1940

Muraine, M 1002

Murati, M 1392

Murdoch, C 1582

Murphy, G 38

Murphy, I 565

Murphy, MFG 1783

Murphy, RA 1469

Murray, LJ 13

Murray, MJ 575

Muss, H 189

Muti, P 1458

Myers, E 28

Myerson, RJ 1654

Myklebust, MP 1719

Mylonakis, N 897

Nabeshima, K 824

Nagai, T 1210

Nagaraju, M 1313

Nagasaka, T 1288

Nagase, S 1267

Nagata, H 104, 1733

Nagata, M 1302, 1322

Nakagawa, K 407, 807, 1210

Nakagawa, M 833

Nakagawa, Y 1322

Nakajima, Y 1191

Nakayama, H 1322

Nakayama, J 824

Nakayama, K 420

Nakayama, M 1615

Nakayama, S 420

Namba, M 1302

Naparstek, E 1708

Naritoku, WY 1224

Nasser, E 272

Natalicchio, MI 1183

Nathan, PD 353

Navarrete, A 612

Navarro, S 89

Nawa, A 1288

Nawrocki, ST 1563

Neal, DE 931

Negrier, S 353

Nelson, WG 602

Németh, M 185

Nevin, RL 602

Newcomb, PA 162

Nexo, E 1850

Ng, K 1235

Ngan, S 498

Nguewa, PA 1608

Nguyen, B-D 945

Nguyen, MN 1874

Nibu, K 393

Nicholson, JC 575

Nicol, AJ 778

Nieda, M 778

Nielsen, B 1218

Nieters, A 1768

Nijman, HW 93

Nishida, Y 1839

Nishimura, T 1693

Nishio, K 407, 1210

Nishio, M 1131

Nishioka, N 1750

Nitta, M 1235

Noda, S 996

Nodin, B 666

Noguera, R 89, 1940

Noh, D-Y 1934

Nohata, N 833

Nonclercq, D 1726

Nonomura, K 1191

Norat, T 1436

Nordenstedt, H 154

Norden-Zfoni, A 112

Normanno, N 1030

Norton, ID 1370

Novelli, MR 1218

Novotny, W 1830

Numasaki, M 1302

Nunn, H 925

Nuovo, G 1023

Nuttall, K 340

Nutting, CM 618, 628, 1804

Nuyten, DSA 44

Nyberg, T 737

O’Brien, L 467, 1230

Ochiai, A 403

Ochiai, T 1733

O’Connor, JPB 139

O’Day, S 346

Offit, K 864

Ogasawara, N 403

O’Grady, A 1487

Ohana, A 1708

Ohashi, Y 1267

Ohlson, N 1436, 1458

Ohtsu, A 403

Ohyanagi, F 1131

Ojima, H 131

Okada, Y 1615

Okamoto, I 407, 807, 1210

Okamoto, K 104, 1733

Okamoto, S 1302

Okamoto, W 407

Okamoto, Y 833

Okano, H 1615

Okano, HJ 1615

Oksefjell, H 890

Olden, K 1750

Oldenburg, RA 1912

Oliver, A 628

Olivier, J 1166

Olson, J 1934

Olsson, C 737

Olwill, SA 1487

Omar, L 18

O’Neill, A 112

Ong, CW 658

Onland-Moret, NC 1436

Opdahl, S 731

Ormiston-Smith, N 460

Ørntoft, TF 552, 1379

Orsini, N 1061

Ortiz, ÁR 1600

Oruzio, D 206

Osako, T 1197

Osipo, C 796

Osler, M 1042

Øster, B 552

Østerlind, K 1042

Ota, K 1322

O’Toole, SA 272

Otsuji, E 104, 1733

Otsuki, Y 420

Otten, JDM 594

Ottensmeier, C 346, 353

Oukrif, D 1218

Overvad, K 1436

Oxley, JD 931

Oya, M 1331

Paap, E 594

Pabst, T 970

Pace, KT 1741

Páez, D 53

Page, E 467

Pajares, MJ 1608

Pakpahan, R 602

Pala, V 1436

Pallis, AG 1

Palmer, RD 575

Pan, D 1012

Pan, L-Z 1012

Pan, Y 1830

Panagiotidi, E 897

Panasci, LC 1342

Pandey, RK 1512

Pandya, K 796

Pankratz, VS 1934

Papachrysanthou, T 897

Papadatos, G 272

Papadopoulos, K 938

Papsdorf, K 445

Paquette, B 534

Parashar, D 766

Paré, L 53

Parikh, A 1759

Parisi, G 327

Park, DJ 413, 760

Parker, GJM 139

Parker, T 467

Parkin, DM 1795

Parma, G 1144

Parmar, MKB 194, 884, 1107

Parry, S 162

Parvinen, I 1388

Pasche, B 640

Pascoe, J 597

Passos, M 1894

Patel, R 760

Patil, M 1252

Patmore, R 1684

Patnick, J 983

Pavlick, A 346

Pavlova, TV 74

Payne, H 1628

Payne, MJ 1252

Peach, M 1830

Peake, MD 746

Pearson, A 1940

Pearson, S-A 1166

Peasland, A 372

Pecorelli, S 1176

Pedersen, L 1776

Peeters, M 1495

Penco, M 1663

Pereira, SP 1370

Pérez, JIA 1934

Perez-Moreno, P 1338

Pervin, S 428

Pesatori, AC 320

Peterlongo, P 1934

Peters, C 961

Peters, GJ 1542

Peto, J 1934

Petricoin, E 1759

Petrova, TV 1346

Petru, E 360

Pfeffer, LM 1874

Pfeffer, SR 1874

Pfeiffer, RM 38

Pfister, C 1811

Pfisterer, J 890, 1144

Pharoah, PP 1934

Pierobon, M 1759

Pierron, G 1940

Pieterse, S 1669

Pietzner, K 1818

Pilavdzic, D 1342

Pio, R 1608

Piqué, JM 870

Piqueras, M 89

Pirozzi, G 1030

Pirson, S 596

Pisano, C 1144

Pitisuttithum, P 28

Planchard, D 847, 1338

Plante, M 1144

Platz, EA 602

Plummer, R 628

Pocock, R 467

Poirier, J 1697

Politis, P 897

Pollak, M 1467

Polláková, V 1533

Ponten, F 565

Pooley, KA 1934

Postmus, PE 1912

Potter, JD 162

Powles, T 766

Prades, J 753

Prendergast, S 353

Pretorius, M 1218

Pritchard, C 1788

Procaccini, V 1663

Pronk, A 1443

Protheroe, AS 1252

Prudnikova, TY 74

Puchtler, G 206

Pujade-Lauraine, E 360, 1144

Purdue, MP 1096, 1772

Puumala, SE 1396

Pylkäs, K 1934

Pylkkänen, L 1388

Qian, W 884

Qiu, H 1522

Raab, I 263

Rabbitt, CA 890

Rabe, KF 673

Rabkin, CS 38

Radkowski, R 773

Rafferty, M 565

Rahman, AA 467

Rahman, M 420

Rahman, MT 420

Raja, FA 884

Ramirez, AJ 18, 1474

Raschle, J 970

Rashid, A 1424

Ratner, E 1176

Ratschiller, D 970

Ravi, R 1295

Ravichandran, D 1825

Ravindranath, A 542

Raymond, E 1640

Rébé C, 366

Reed, M 189, 1260

Reed, N 360

Regier, D 606

Reichardt, P 505

Reid, A 1409

Reid, TD 842

Reiman, A 586

Reiniš, M 1533

Ren, J 1905

Reñé, JM 870

Rennert, G 562

Reynolds, P 1396

Riaz, SP 170

Ribeiro, A 1940

Riboli, E 1436

Ricart, A 938

Ricevuto, E 1663

Rich, AL 746

Richards, J 346

Richards, S 170

Richon, C 1352

Richter, B 1144

Richter, GHS 596

Richter, R 1818

Rigal, O 1811

Rijlaarsdam, MA 854

Rimmer, Y 766

Rinaldi, S 1436

Ring, A 189, 1260

Rios, M 1480

Ristamäki, R 255

Ristimäki, A 989

Rittweger, K 58

Robert, C 353

Roberts, C 139

Roberts, J 796

Roberts, JD 1750

Robien, K 1430

Robinson, A 1260

Robinson, D 170

Robinson, MC 931

Robson, CN 1362

Robson, SC 469

Rocha-Lima, CM 44

Roché, H 1480

Rodríguez, L 1436

Rodríguez, MJ 1608

Roelens, J 877

Rogov, YI 1934

Rogowski, A 796

Rojo, F 814

Roma, C 1030

Roman, E 1684

Romano, S 1663

Romieu, I 1436

Romundstad, PR 731

Roncalli, M 1542

Rosario, DJ 931

Rose, C 1676

Rose, CJ 139

Rothman, N 1096, 1443, 1772

Rothmann, F 505

Rots, MG 312

Rotunno, M 320

Rovira, A 814

Rowinsky, EK 938

Rowling, E 372

Roy, D 320

Roy, S 1654

Royer, R 534

Royle, JA 1076

Rubagotti, M 320

Rubie, H 1940

Ruggero, D 329

Ruggieri, C 1183

Ruiters, MHJ 312

Ruiz-Ponte, C 870

Russo, GL 221

Russo, M 221

Ruterbusch, JJ 1096, 1772

Rutherford, TJ 1176

Ryan, D 565

Ryba, N 760

Saah, A 28

Sabatier, R 304

Sabel, MC 961

Sacerdote, C 1436

Saha, B 1224

Sahebjam, S 1342

Saijo, N 1267

Saintigny, P 382

Saito, H 1267

Sakai, H 1267

Sakai, K 807, 1210

Sakurai, D 833

Salazar, J 53

Salazar, R 1646

Salès, F 1726

Salido, M 814

Saló, J 870

Salomon, DS 1030

Salto-Tellez, M 658

Saltz, L 58

Samuel, S 1759

Sanchez, B 1608

Sánchez, M-J 1436

Sandanayake, NS 1370

Sandler, A 1295

Sangrajrang, S 1934

Sankila, R 1069

Sanner, K 337, 694

Santin, AD 1176

Santoro, A 1542

Sanyaolu, LN 842

Sarkaria, J 372

Sartelet, H 1697

Sasajima, Y 698

Sasieni, PD 460, 1795

Sathya, S 953

Saunders, E 1230

Savage, SA 1772

Sawada, T 996

Sawyer, E 467, 1230, 1934

Schadendorf, D 346

Scheithauer, W 58

Scherpen, FJG 1856

Schleiermacher, G 1940

Schmidt, MK 1934

Schmidt, W 263

Schmitt, M 1864

Schmutzler, RK 1934

Schneider, C 1002

Schoemaker, MJ 911

Schultz, H 673

Schulze, M 206

Schürmann, P 1934

Schuster, E 1593

Schütze, M 1436

Schüz, J 1042, 1069

Schwalbe, EC 575

Schwaner, I 505

Schwartz, G 938

Schwartz, KL 1096, 1772

Schwartz, PE 1176

Scott, CJ 1487

Scott, KJ 787

Scotting, PJ 575

Sebastian, P 618

Sebio, A 53

Seddon, AM 1554

Seelinger, M 263

Sehouli, J 890, 1593, 1818

Seidel, C 1635

Seiki, M 824

Seki, N 833

Selby, P 22

Selmer, R 157

Semaan, A 493

Serin, D 1480

Serrano, S 814

Seshadri, M 1512

Sethi, N 1805

Severi, G 438, 1934

Seymour, MT 194

Sguotti, C 1646

Shaikh, H 1414

Shamash, J 766

Shao, R 1203

Sharif, A 466

Sharrocks, AD 124

Shashurin, A 1295

Shaw, PHS 649

Sheahan, K 565

Shebl, FM 1424

Shelton, J 460

Shen, M-C 1424

Shi, J 658

Shibata, H 212

Shibata, K 1288

Shibata, T 131, 698

Shigemura, K 393

Shimada, H 1302

Shimada, K 1191

Shimizu, H 131

Shimokawa, H 1465

Shimozuma, K 1273

Shimura, T 1885

Shinohara, M 1322

Shiozaki, A 104, 1733

Shipe-Spotloe, J 773

Shirakawa, H 1331

Shirakawa, T 393

Shiroiwa, T 1273

Shirotake, S 1331

Shmulevitz, M 1012

Sho, M 1191

Shore, RE 1458

Shu, X-O 1443

Sieri, S 1458

Sigalotti, L 327

Silasi, D-A 1176

Silcocks, PB 460

Silva, CL 1894

Simes, RJ 360, 1144

Simmonds, P 1260

Šímová, J 1533

Simpson, K 340

Sinclair, J 1370

Singer, S 445

Singh, R 428

Sinha, R 1096

Siu, LL 44

Skarstein, A 1719

Skog, M 1346

Skorpen, F 1244

Slade, K 340

Slominski, AT 1874

Smid, K 1542

Smith, A 1684

Smith, G 246

Smith, P 687

Smith, RC 1370

Sokoll, LJ 602

Solca, F 1554

Solheim, TS 1244

Song, E 146

Soo, K-C 945

Sorensen, BS 1850

Sørensen, HT 881, 1776

Sorg, RV 961

Soria, JC 847, 1338

Sorio, R 360

Sørlie, T 9

Southey, MC 1934

Spagnuolo, C 221

Spector, LG 1392, 1396

Speiser, P 1593

Spielmann, M 1480

Srinivasan, P 1295

Srinivasan, V 586

Srivastava, S 658

Staines, A 1768

Stanczyk, FZ 1424

Stanimirovic, D 1697

Stanley, RA 746

Starink, TM 1912

Stark, D 766

Stary, S 263

Stauch, M 206

Steding-Jessen, M 1042

Steele, RJC 246

Steffen, A 1436

Steiger, H-J 961

Steineck, G 737

Štěpánek, I 1533

Stevens, KN 1934

Stevenson, MR 1487

Steverlynck, C 1002

Stewart, R 1741

Stewart-Brown, S 467

Stifano, G 1663

Stiller, CA 1783

Stintzing, S 206

Stoop, H 854

Strasser, F 1244

Stratford, IJ 628

Straume, O 9

Strauss, UP 353

Strizzi, L 1030

Stummer, W 961

Sturla, L-M 1235

Sudhagar, S 953

Sugiyama, T 1267

Sukhamwang, N 1224

Sukhatme, VP 112

Sulecki, M 773

Sullivan, T 1669

Sundström, J 255

Sung, F-C 1419

Sung, L 606

Surjan, R 640

Sutcliffe, S 602

Sutherland, RL 272

Suzuki, H 1885

Suzuki, K 778

Suzuki, S 1288

Swart, AM 884

Sweeney, KJ 1625

Swensen, RE 493

Swerdlow, AJ 911

Syed, S 44

Székely, B 185

Szutowicz, E 1

Taal, BG 1173

Tabernero, J 1646

Tagawa, M 1302

Tahara, M 403

Takeshita, H 104, 1733

Talbot, DC 1252

Tamimi, H 493

Tamura, D 1210

Tan, B 1244

Tan, BR 1654

Tan, CL 575

Tan, E 1646

Tan, S-B 945

Tan, SS 1362

Tanaka, F 1465

Tanaka, K 1210

Tanaka, N 1331

Tanaka, T 1322

Tang, J-L 975, 1927

Tang, W 146, 320, 1574

Tang, W-Y 65

Tang, Y 1905

Taniguchi, H 1733

Tanizaki, J 807

Tarhini, A 773

Tata, LJ 746

Taverna, P 1563

Tawbi, HA 773

Taylor, AM 586

Taylor, CR 1224

Taylor, M 1252

Teichmann, M 263

Temam, S 618

ter Elst, A 1856

Terpstra, P 312

Teuffel, O 606

Therriault, H 534

Theunis, A 1726

Thibodeau, SN 162

Thiel, U 596

Thomas, D 911

Thomas, F 1654

Thomas, G 1260

Thomas, GD 1934

Thomas, L 1352

Thompson, AM 1627

Thompson, DJ 586

Thompson, G 139

Thompson, JA 346

Thomson, CS 460

Thomson, TM 1600

Thornhill, MH 1582

Thorsen, K 552

Thurnham, A 1474

Thuss-Patience, PC 505

Tian, S 1905

Tian, WJ 890

Tien, H-F 975, 1927

Tierney, JF 1107

Timmerman, D 187

Timmermans, M 854

Timms, JF 1370

Tisdale, MJ 83

Tjønneland, A 1436

Todeschini, P 1176

Toi, M 1693

Tokuyama, H 778

Tolcher, AW 938

Tollenaar, RAEM 281, 1934

Tomlinson, IPM 870, 1934

Tømte, E 296

Tonini, GP 1940

Toniolo, P 1458

Tozzi, F 1759

Trakultivakorn, H 1224

Tran, A 428

Tran, L 428

Travier, N 1436

Trefzer, U 346

Trichopoulou, A 1436

Trink, B 1295

Troiani, T 382

Tropé, CG 890

Truitt, ML 329

Tsakonas, G 897

Tsao-Wei, D 1224

Tsavaris, N 897

Tsay, W 975

Tseng, M-H 975

Tsilidis, KK 1436

Tsuchihara, K 403

Tsuda, H 698

Tsujiura, M 104, 1733

Tsutsumi, S 1885

Tuccillo, C 382

Tucker, L 18

Tuckey, RC 1874

Tulunay, G 360

Tumino, R 1436

Turnill, N 340

Turrell, G 1039

Tverdal, A 157

Tweddle, DA 1940

Twelves, CJ 194

Tymrakiewicz, M 1230

Uhlar, C 346

Uhlén, M 666

Ulhøi, BP 1379

Ulmer, H 1593

Umezu, T 1288

Unger, C 263

Unwin, L 565

Urakawa, H 1839

Uramoto, H 1465

Urban, P 1646

Urman, R 428

Vachon, CM 1934

Valteau-Couanet, D 1352

van Blankenstein, M 200

van de Nieuwenhof, HP 1279

van den Einden, LCG 1279

van der Gun, BTF 312

van der Zee, AGJ 1279

van Doorn, MBA 1912

van Duijnhoven, FJB 1436

Van Gorp, T 187

van Grieken, NCT 1912

van Herk, HADM 854

Van Kempen, L 1726

van Leenders, GJLH 854

van ‘t Veer, LJ 281, 1934

van Moorselaar, RJA 1912

van Os, TA 1912

van Ravesteyn, NT 1082

van Roy, N 1940

van Schoor, G 594

van Spaendonck-Zwarts, KY 1912

van Steensel, MAM 1912

van Velthuysen, M -L F 281

van Waesberghe, J-HTM 1912

Vannier, JP 1002

Varma, M 931

Varughese, J 1176

Vasse, M 1002

Vassilev, LT 1012

Vatten, LJ 731

Vehling-Kaiser, U 206

Velasquez, L 44

Venning, G 1107

Verbeek, ALM 594

Vergnenegre, A 1123

Vergote, I 187, 1144, 1593

Verhagen, MM 586

Verwaal, VJ 281

Verweij, J 938

Veyret, C 1480

Vicente, V 612

Vielh, P 847, 1338

Viens, P 304

Vijai, J 864

Vilella, À 870

Villamon, E 1940

Villano, JL 1414

Villaruz, L 773

Vimond, N 847

Vincent, A 281

Vinogradova, Y 452

Viola, K 263

Violette, SM 1582

Viraswami-Appanna, K 1646

Vitek, P 1646

Viverette, F 773

Vo, TPN 263

Vodermaier, A 1814

Volinia, S 1023

Vollmer, E 673

Von Behren, J 1396

Vonach, C 263

Vorgias, G 897

Vrieling, A 1151

Vuocolo, S 28

Wacholder, S 1096, 1772

Wacker, B 938

Wada, W 1885

Wagner, R 534

Wagner, U 1144

Wainberg, ZA 760

Waldenström, A-C 737

Walk, R 1295

Walker, DA 575

Walker, K 1451

Walker, MJ 1023

Wallner, S 231

Walsh, K 246

Walters, K 1920

Wang, B-S 1424

Wang, E 320

Wang, H-J 760

Wang, J 146, 1934

Wang, L-Z 372

Wang, R 1430

Wang, T 658

Wang, X 1934

Wangefjord, S 666

Wang-Gohrke, S 1934

Ward, AM 475

Ward, RL 1166

Wareham, N 1436

Warren, AY 931

Wasa, J 1839

Wason, J 766

Watson, Y 139

Weber, B 1850

Webster, GJ 1370

Weemaes, CM 586

Weidenaar, AC 1856

Weidlich, S 246

Weikert, S 1635

Weinreb, PH 1582

Welge, J 65

Welinder, C 666

Wen, PY 112

Wentzensen, N 877

West, EJ 787

Westermarck, J 989

Wheeler, K 1940

Whitby, D 1768

Whitcher, BJ 139

White, ID 903

White, J 766

White, NMA 1741

Wiedig, M 1726

Wiezorek, J 1495

Wikström, I 337, 694

Wilander, E 337, 694

Wilderäng, U 737

Wildiers, H 189

Wilkinson, RA 467, 1230

Wilkinson, S 1054

Willemsen, MA 586

Williams, GT 842

Williams, MV 766

Williamson, J 911

Willis, S 1574

Wilson, RH 1487

Win, AK 162

Win, K-M 945

Wing, R 606

Winqvist, R 1934

Winship, I 162

Winterdahl, M 1850

Winton, DJ 1253

Witt, K 938

Wolf, A 1593

Wolf, CR 246

Wolk, A 441, 1061

Wollschlaeger, KM 890

Wong, R 58

Wood, L 938

Wootton, LL 586

Wu, C-P 513

Wu, G 146

Wu, HK 112

Wu, S-J 975

Wu, T 65

Wyke, SM 83

Xia, L 1759

Xiao, Z 146

Xicola, RM 870

Xing, Y 890

Xu, F 1495

Xu, J 146

Xu, K 146

Xu, R 1830

Xu, Z 1905

Xue, A 1370

Xue, S 1443

Yajima, T 1885

Yamada, Y 1210

Yamaguchi, N 1615

Yamashita, K 1197

Yamato, I 1191

Yan, B 658

Yan, L 1905

Yan, W 1203

Yanagihara, K 407, 1210, 1693

Yang, G 1443

Yao, M 975, 1927

Yashiro, M 996, 1522, 1750

Yeasmin, S 420

Yeoh, KG 658

Yeole, BB 723

Yeung, E 1469

Yokokawa, K 778

Yong, WP 658

Yoo, K-Y 1934

Yoshida, R 1322

Yoshikawa, D 131

Yoshino, H 833

Yoshino, T 403

Yoshitake, Y 1322

Yoshiwara-Wakabayashi, E 38

Young, B 1574

Young, GP 162

Young, JP 162

Young, L 1224

Young, LS 118

Yousef, GM 1741

Youssef, YM 1741

Yu, F 146

Yu, K 1424

Yu, MC 1430

Yuan, J-M 1430

Yuen, H-F 542

Zabarovsky, ER 74

Zabel, P 673

Zaineddin, AK 1151

Zalutsky, JV 1934

Zanesco, T 640

Zang, RY 890

Zeillinger, R 1593

Zeimet, AG 1593

Zeleniuch-Jacquotte, A 1458

Zellmann, K 206

Zenilman, JM 602

Zhang, B-H 1424

Zhang, J 1495

Zhang, L 112

Zhang, M 1089

Zhang, X 945, 1522

Zhao, Y 320

Zhao, Z 1495

Zheng, W 1443

Zhivkova-Galunska, M 288

Zhong, B 346

Zhou, X 1495

Zhuo, L 1839

Zierhut, H 1392

Zimmerer, JM 1023

Zimmerman, J 640

Zimmerman, LJ 1759

Zinn, PO 1235

Ziogas, A 1934

Zissel, G 673

Zucali, PA 1542

Zurita, AJ 112

Zwerenz, R 445

Zylis, D 1436

